# Data on the risk perceptions of beach water safety in coastal Georgia

**DOI:** 10.1016/j.dib.2018.04.113

**Published:** 2018-05-05

**Authors:** Jeff Jones, Aslī Aslan, Rakhi Trivedi, Maria Olivas, Mikayla Hoffmann

**Affiliations:** Jiann-Ping Hsu College of Public Health, Georgia Southern University, PO Box 8015, Statesboro, GA 30460, USA

## Abstract

These data reflect the perceptions of beach water quality drawn from a convenience sample of 238 visitors to Georgia (USA) beaches collected in June–July 2017 and are related to the research article entitled “Water quality and the perception of risk: a study of Georgia, USA, beachgoers” (Jones et al., 2018) [1]. Data were collected both via an online survey distributed through Facebook and through in-person questionnaires collected directly on the beaches.

## Specifications Table

**Table:** 

Subject area	*Public Health*
More specific subject area	*Environmental Health*
Type of data	*Tables, Figure (Map)*
How data was acquired	*Survey*
Data format	*Analyzed*
Experimental factors	*The data were analyzed by various demographic strata (residency, age, sex, race, and education).*
Experimental features	*The relationship between demographic characteristics and the perception of what constitutes clean or polluted beach water were determined.*
Data source location	*Jiann-Ping Hsu College of Public Health, Georgia Southern University, Statesboro, Georgia, USA*
Data accessibility	*With this article*

## Value of the data

• These data could be useful in comparing perceptions of beach water quality among beachgoers in other states and countries.
• Because these data were collected during the busy summer swimming season, these data could be useful in comparing perceptions of beach water quality among beachgoers visiting beaches in other seasons.
• Data were collected in such a way as to stratify among full-time residents, part-time residents, and visitors. These data may thus be useful to researchers comparing coastal perceptions between residents and visitors.

## 1 Data

This dataset presents information on the varied perceptions of what constitutes water quality including whether respondents view the absence of waterborne pathogens as the primary feature of water quality at tourist beaches. Data also include which illnesses associated with waterborne pathogens are perceived as linked to unclean water by respondents. Table 1, Table 2, Table 3, Table 4, Table 5, Table 6, Table 7, Table 8, Table 9, Table 10 present information by different demographic characteristics. The results from this dataset are presented in Jones et al. [1].

**Table 1:** Illnesses by sex.

**In respondent׳s opinion, this health risk is associated with recreational activities in polluted beach water**	**Female (%)**	**Male (%)**
No Risks	1.1	3.2
Upset Stomach/Diarrhea	78.7	80.6
Swimmer׳s Ear	51.1	58.1
Red, Itchy Eyes/Eye Infections	68.4	79.0
Wound Infections	78.7	82.3

**Table 2:** Illnesses by education.

**In respondent׳s opinion, this health risk is associated with recreational activities in polluted beach water**	**Without a 4-year college degree (%)**	**With a 4-year college degree (%)**
No Risks	0.0	2.3
Upset Stomach/Diarrhea	69.4	83.0
Swimmer׳s Ear	53.2	52.8
Red, Itchy Eyes/Eye Infections	71.0	71.6
Wound Infections	83.9	78.4

**Table 3:** Illnesses by income.

**In respondent׳s opinion, this health risk is associated with recreational activities in polluted beach water**	**Income below U.S. median household income (%)**	**Income above U.S. median household income (%)**
No Risks	0.0	2.2
Upset Stomach/Diarrhea	75.8	83.8
Swimmer׳s Ear	50.5	54.4
Red, Itchy Eyes/Eye Infections	70.3	71.3
Wound Infections	81.3	77.9

**Table 4:** Illnesses by race.

**In respondent׳s opinion, this health risk is associated with recreational activities in polluted beach water**	**White (%)**	**Racial minorities (aggregate of all non-white respondents) (%)**
No Risks	1.0	8.7
Upset Stomach/Diarrhea	80.2	73.9
Swimmer׳s Ear	52.7	56.5
Red, Itchy Eyes/Eye Infections	70.0	73.9
Wound Infections	79.7	73.9

**Table 5:** Illnesses by residency.

**In respondent׳s opinion, this health risk is associated with recreational activities in polluted beach water**	**Visitor (%)**	**Part-year resident (%)**	**Year-round resident (%)**
No Risks	1.4	0.0	3.6
Upset Stomach/Diarrhea	77.1	85.2	80.0
Swimmer׳s Ear	59.0	48.1	41.8
Red, Itchy Eyes/Eye Infections	73.6	63.0	70.9
Wound Infections	81.3	85.2	74.5

**Table 6:** Perceptions by sex.

**This factor BEST explains what clean beach water means to a respondent**	**Female (%)**	**Male (%)**
No disease-causing pathogens in the water	52.3	38.7
No trash	20.3	32.3
Clear or colorless water	18.6	21.0
Odorless water	8.1	8.1
No wildlife	0.6	0.0

**Table 7:** Perceptions by education.

**This factor BEST explains what clean beach water means to a respondent**	**Without a 4-year college degree (%)**	**With a 4-year college degree (%)**
No disease-causing pathogens in the water	35.0	53.4
No trash	23.3	23.9
Clear or colorless water	26.7	16.5
Odorless water	15.0	5.7
No wildlife	0.0	0.6

**Table 8:** Perceptions by income.

**This factor BEST explains what clean beach water means to a respondent**	**Income below U.S. median household income (%)**	**Income above U.S. median household income (%)**
No disease-causing pathogens in the water	56.3	48.0
No trash	27.1	22.6
Clear or colorless water	8.3	20.9
Odorless water	8.3	7.9
No wildlife	0.0	0.6

**Table 9:** Perceptions by race.

**This factor BEST explains what clean beach water means to a respondent**	**White (%)**	**Racial minorities (aggregate of all non-white respondents) (%)**
No disease-causing pathogens in the water	50.2	39.1
No trash	22.0	34.8
Clear or colorless water	19.5	13.0
Odorless water	7.8	13.0
No wildlife	0.5	0.0

**Table 10:** Perceptions by residency.

**This factor BEST explains what clean beach water means to a respondent**	**Visitor (%)**	**Part-year resident (%)**	**Year-round resident (%)**
No disease-causing pathogens in the water	43.7	63.0	61.8
No trash	23.9	14.8	25.5
Clear or colorless water	21.1	18.5	7.3
Odorless water	10.6	3.7	5.5
No wildlife	0.7	0.0	0.0

## 2 Experimental design, materials and methods

Researchers collected these data using a quantitative survey offered either online via recruiting on Facebook or, primarily, through in-person collection of paper surveys from visitors to Georgia beaches in June–July 2017. In-person data collection focused on two major Georgia recreational beaches (Tybee Island and Jekyll Island) during peak swimming season. This study was approved by the Georgia Southern University Institutional Review Board with participants’ consent required to complete the survey. Data were analyzed using IBM SPSS 23 (IBM, Armonk, NY) and ArcMap 10.4.1 (Esri, Redlands, CA).

Data variables include:

• Consent (all respondents have given consent).
• Description of which factor most represent clean water to a respondent.
• 5 variables asking whether a respondent perceives as not (0) or having (1) as association with polluted water.
• Age.
• Sex.
• Hispanic ethnicity.
• Race (original answer).
• Race2 (dichotomized to white (0) or minority (1)).
• Income (dichotomized as income below U.S. median household income (0) or higher (1)).
• Education (dichotomized as without a 4-year college degree (0) or with one (1)).
