# Supplementary material for: Data on the risk perceptions of beach water safety in coastal Georgia
Source: Data Brief. 2018 May 5;19:312–6. doi: 10.1016/j.dib.2018.04.113 (PMC5992957; doi:10.1016/j.dib.2018.04.113)
Supplement: Supplementary file 1 — Supplementary material [file mmc1.docx]

No conflicts of interest to report
